# Thyroid crisis caused by metastatic thyroid cancer: an autopsy case report

**DOI:** 10.1186/s12902-021-00875-7

**Published:** 2021-10-24

**Authors:** Kai Takedani, Masakazu Notsu, Naoko Adachi, Sayuri Tanaka, Masahiro Yamamoto, Mika Yamauchi, Naotake Yamauchi, Riruke Maruyama, Keizo Kanasaki

**Affiliations:** 1 Department of Internal Medicine 1, Faculty of Medicine, Shimane University, 89-1 Enya-cho, Izumo, Shimane 693-8501 Japan; 2 Department of Pathology, Faculty of Medicine, Shimane University, 89-1 Enya-cho, Izumo, Shimane 693-8501 Japan; 3 Department of Pathology, Faculty of Medicine, University of Toyama, 2630 Sugitani, Toyama, Toyama 930-0194 Japan

**Keywords:** Thyroid crisis, Follicular thyroid carcinoma, Anaplastic thyroid carcinoma, Metastasis

## Abstract

**Background:**

Thyroid crisis is a life-threatening condition in thyrotoxic patients. Although differentiated thyroid cancer is one of the causes of hyperthyroidism, reports on thyroid crisis caused by thyroid cancer are quite limited. Here, we describe a case of thyroid crisis caused by metastatic thyroid cancer.

**Case presentation:**

A 91-year-old woman was admitted to our hospital because of loss of appetite. Two years prior to this hospitalization, she presented with subclinical thyrotoxicosis and was diagnosed with histologically unidentified thyroid cancer with multiple metastases, and she refused aggressive medical interventions. On admission, she exhibited extreme thyrotoxicosis, and the presence of fever, severe tachycardia, impaired consciousness, and heart failure revealed the presence of thyroid crisis. All thyroid autoantibodies were negative. Multidisciplinary conservative treatment was initiated; however, she died on the fifth day after admission. Autopsy revealed the presence of primary anaplastic thyroid carcinoma and multiple metastatic foci arising from follicular thyroid carcinoma. Both primary and metastatic follicular thyroid carcinoma likely induced thyrotoxicosis, which could have been exacerbated by anaplastic thyroid carcinoma.

**Conclusions:**

Even though the trigger of thyroid crisis in this patient is not clear, the aggravated progression of her clinical course suggests that careful monitoring of thyroid hormones and appropriate intervention are essential for patients with thyroid cancer.

## Background

Thyroid crisis is a life-threatening condition requiring emergency treatment in thyrotoxic patients [1, 2]. Patients with thyroid crisis exhibit multiple organ failure induced by the disruption of haemodynamics due to excess thyroid hormone and have a mortality rate higher than 10% in Japan [1]. Graves’ disease is the most common cause of both hyperthyroidism and thyroid crisis [1, 3]. Often, individuals with thyroid crisis have triggers for the onset of thyroid crisis. Although differentiated thyroid cancer is one of the causes of hyperthyroidism, [4] reports on thyroid crisis caused by thyroid cancer are quite limited.

Here, we present a case of uncontrollable thyroid crisis caused by thyroid cancer with multiple metastases.

## Case presentation

**Fig. 1:**
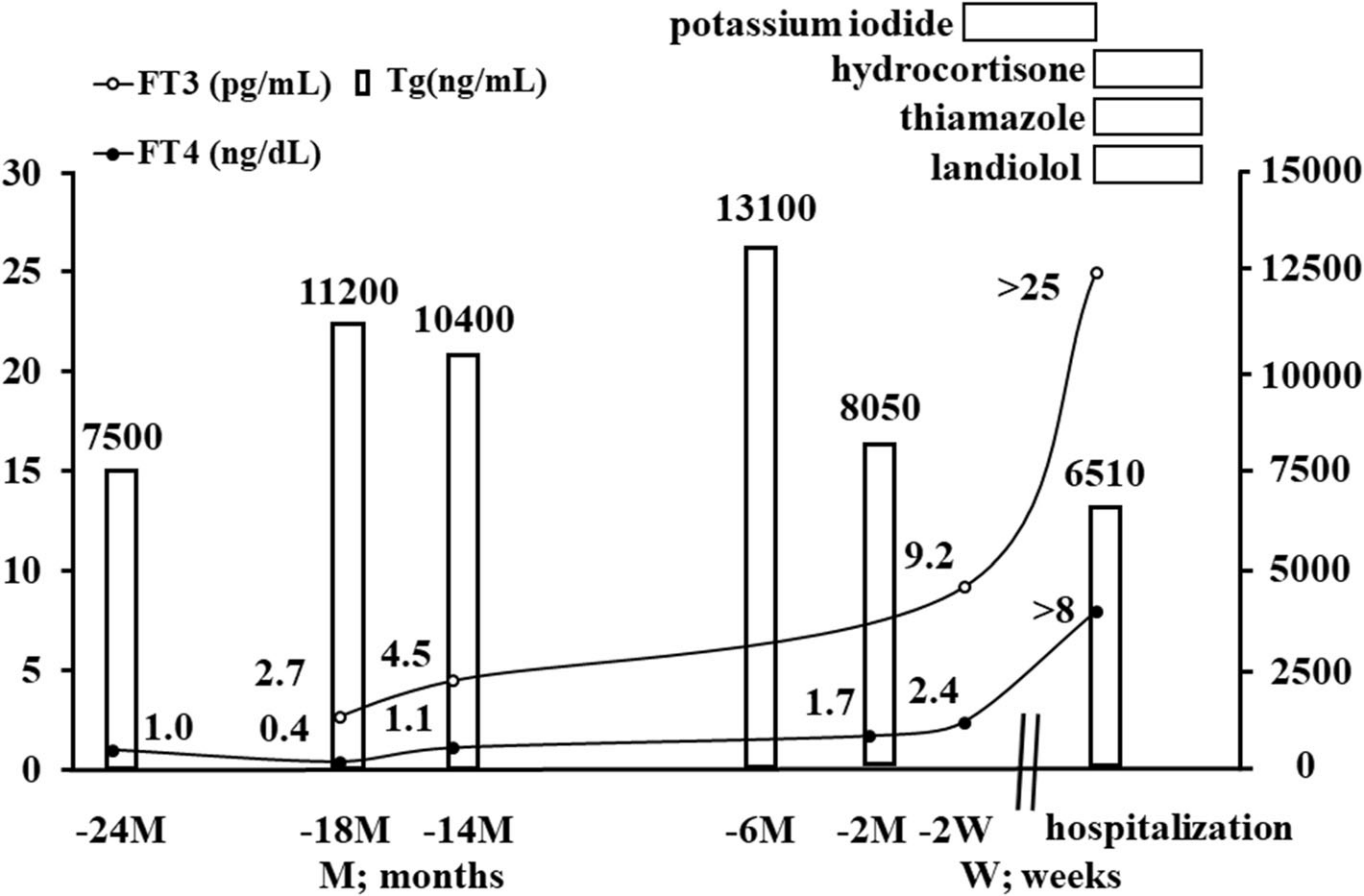
Clinical course. When the patient was diagnosed with a thyroid tumour, her TSH was under the detection limit, and her thyroid hormone levels were normal; she had subclinical thyrotoxicosis. Her thyroid hormones worsened gradually until 2 weeks before admission; however, thyrotoxicosis rapidly deteriorated in the last 2 weeks. She also had a high Tg level at the first visit, which peaked 2 months before admission and then decreased

A 91-year-old woman was admitted to Shimane University Hospital because of loss of appetite. Two years prior to this hospitalization, at the age of 89, computed tomography (CT) scans incidentally revealed a 55 mm tumour in the right lobe of the thyroid gland, mediastinal lymphadenopathy, and multiple pulmonary nodules. Fine-needle aspiration cytology demonstrated nuclear grooves in the tumour, which formed small follicular structures, but intranuclear cytoplasmic inclusion was not observed. Cytology could not specify the type of thyroid cancer. Due to her age, she did not wish to receive aggressive management to treat the histologically unidentified thyroid cancer with multiple metastases. Her thyroid-stimulating hormone (TSH) was under the detection limit, and she had subclinical thyrotoxicosis (free thyroxine (FT4) 1.0 ng/dL) with high thyroglobulin (Tg) levels (Fig. 1). She did not have any symptoms. We discussed the risks and benefits associated with anti-thyroid treatment with her and her family, and they chose no specific treatment for her thyrotoxicosis. Until 2 months prior to hospitalization, her condition was unremarkable. Two weeks before admission, however, she demonstrated overt thyrotoxicosis (free triiodothyronine (FT3) 9.2 pg/mL, FT4 2.4 ng/dL, and FT3/FT4 ratio 3.8) without overt symptoms. She was prescribed potassium iodide (KI) (50 mg); however, her general status worsened to include loss of appetite. She was hospitalized to improve her general status and to treat thyrotoxicosis.

Six months after the thyroid tumour was diagnosed, she experienced a pathological left hip fracture due to bone metastasis. She also had a history of cerebral infarction treated with an antiplatelet drug. Her activities of daily living were generally suitable before the emergency admission.

Her height was 143 cm, her body weight was 29.3 kg, and her body mass index was 14.3 kg/m^2^. The Glasgow coma scale score was E4V4M6. Her blood pressure was 167/100 mmHg, pulse rate was 160/min, body temperature was 38.4 °C, oxygen saturation (SpO_2_) was 97% (room air), and respiratory rate was 26/min. Her anterior neck was markedly swollen without overt pain. The other physical findings were unremarkable except for mild pitting oedema of the bilateral lower extremities.

**Fig. 2:**
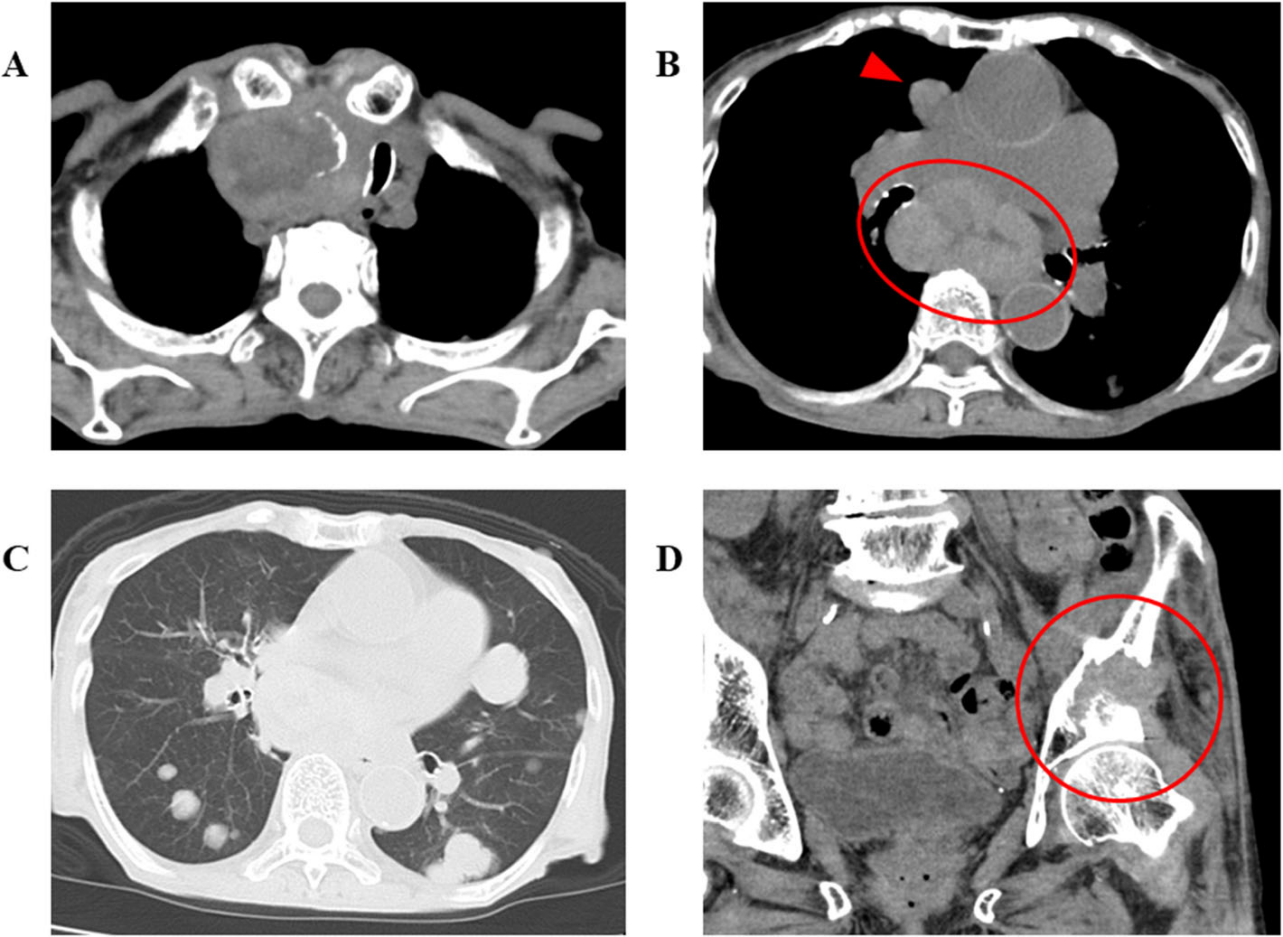
Computed tomography scan. **A**: The thyroid was markedly swollen with calcification. Tracheal deviation was identified. **B**: The hilar and mediastinal lymph nodes were swollen. **C**: Multiple nodules were identified in both lungs. **D**: A pathological left hip fracture was identified

**Fig. 3:**
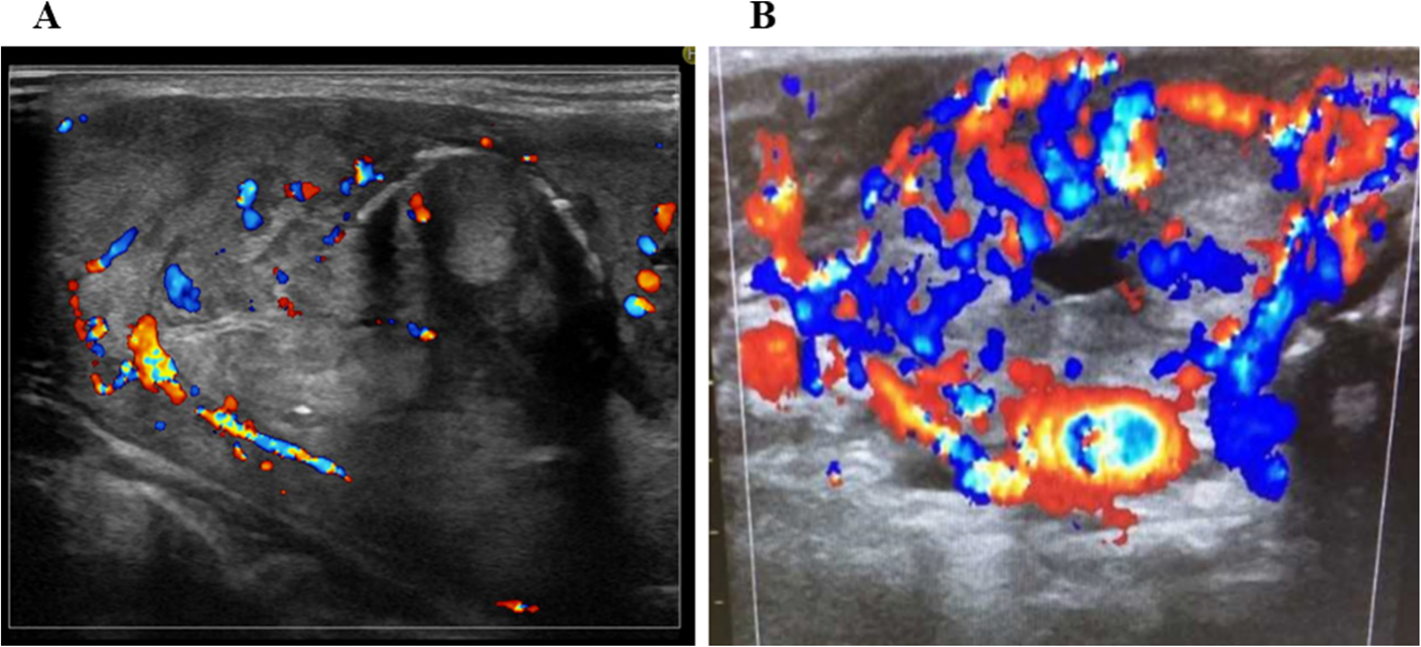
Thyroid ultrasonography. **A** Two years before hospitalization. A large tumour was revealed in the right lobe with calcification and slight blood flow. **B** At the time of hospitalization. Diffuse enlargement of the right lobe with increased blood flow was revealed

The laboratory findings on admission are shown in Table 1. She had extreme thyrotoxicosis above the sensitivity limits (TSH < 0.003 μU/mL, FT3 > 25 pg/mL, FT4 > 8 ng/dL). The presence of fever, severe tachycardia, impaired consciousness, and heart failure suggested thyroid crisis. TSH receptor antibody and Hashimoto thyroiditis-related antibodies were all negative, suggesting that her thyrotoxicosis was caused by thyroid cancer or destructive thyroiditis. A CT scan identified an enlarged thyroid tumour and metastases (Fig. 2). Ultrasound revealed diffuse enlargement of the right lobe of the thyroid gland with increased blood flow (Fig. 3). An electrocardiogram showed severe sinus tachycardia without atrial fibrillation. Echocardiography revealed diffuse asynergy, suggesting takotsubo cardiomyopathy, likely due to the aberrant sympatho-adrenergic activation induced by thyrotoxicosis. The ejection fraction was 22%. The presence of infection was excluded by physical examination, CT images acquired at the time of hospitalization and only minor elevation of an inflammatory marker. Human chorionic gonadotropin (HCG)-induced hyperthyroidism was also excluded.

**Table 1:** Baseline laboratory data

Parameter	Observed	Reference range
Venous blood gas analyses		
pH	7.28	7.35–7.45
pCO_2_, mmHg	34	35–48
HCO_3_^−^, mEq/L	15	21–28
lactic acid, mg/dL	22	4.5–13.5
Urinalysis		
pH	6.0	4.5–7.5
blood	±	–
protein	2+	–
ketone	2+	–
I/Cr, μg/gCr	27,120	200–1000
Complete blood count		
WBC,/μL	12,730	3300–8600
Neutro, %	87	40–75
Hb, g/dL	9.9	11.6–14.8
Plt,/μL	12.0 × 10^4^	15.8–34.8 × 10^4^
Serum characteristics		
Alb, g/dL	3.4	4.1–5.1
T-Bil, mg/dL	0.7	0.4–1.5
AST, IU/L	26	13–30
ALT, IU/L	28	7–23
LDH, IU/L	193	124–222
ALP, IU/L	152	106–322
CK, IU/L	93	41–153
CK-MB, ng/mL	10.6	< 3.7
TNI, ng/mL	1.09	< 0.04
T-chol, mg/dL	95	142–248
HbA1c, %	5.3	4.9–6.0
BUN, mg/dL	28	8–20
Cr, mg/dL	0.54	0.46–0.79
Na, mEq/L	142	138–145
K, mEq/L	4.1	3.6–4.8
Cl, mEq/L	110	101–108
cCa, mg/dL	8.9	8.8–10.1
CRP, mg/dL	2.4	< 0.03
PCT, ng/mL	0.06	< 0.50
BNP, pg/mL	2796	< 20
FT3, pg/mL	> 25	2.1–3.8
FT4, ng/dL	> 8.0	0.8–1.5
TSH, μU/mL	< 0.003	0.50–3.00
TRAb, IU/L	< 0.9	< 2.0
TSAb, %	114	< 120
Tg-Ab, IU/mL	< 5.0	< 5.0
TPO-Ab, IU/mL	< 3.0	< 3.0
Tg, ng/mL	6510	< 33.7
HCG, mIU/mL	< 1.0	< 2.7

*I* iodide, *Cr* creatinine, *WBC* white blood cell, *Neutro* neutrophils, *Hb* haemoglobin, *Plt* platelet, *Alb* albumin, *T-Bil*, total bilirubin, *AST* aspartate transaminase, *ALT* alanine aminotransferase, *LDH* lactate dehydrogenase, *ALP* alkaline phosphatase, *CK* creatine kinase, *TNI* troponin i, *T-chol* total cholesterol, *HbA1c* haemoglobin A1c, *BUN* blood urea nitrogen, *Na* sodium, *K* potassium, *Cl* chlorine, *cCa* corrected calcium, *CRP* C-reactive protein, *PCT* procalcitonin, *BNP* brain natriuretic peptide, *FT3* free triiodothyronine, *FT4* free thyroxine, *TSH* thyroid-stimulating hormone, *TRAb* TSH receptor antibody, *TSAb* thyroid stimulating antibody, *Tg-Ab* anti-thyroglobulin antibody, *TPO-Ab* anti-myeloperoxidase antibody, *Tg* thyroglobulin, *HCG* human chorionic gonadotropin

Despite treatment with KI, her thyrotoxicosis worsened. Considering possible augmentation of her thyrotoxicosis by the administered iodide, the KI was discontinued. Treatment with intravenous hydrocortisone and oral thiamazole was initiated; however, due to her severe illness, the oral administration of thiamazole was not possible. The maximum dose of landiolol also failed to manage her tachycardia. Her condition deteriorated progressively, and she died on the fifth day of hospitalization.

After a discussion with her family, an autopsy was performed.

### <Thyroid> 110 g, 7.6 × 6.4 × 3.0 cm

**Fig. 4:**
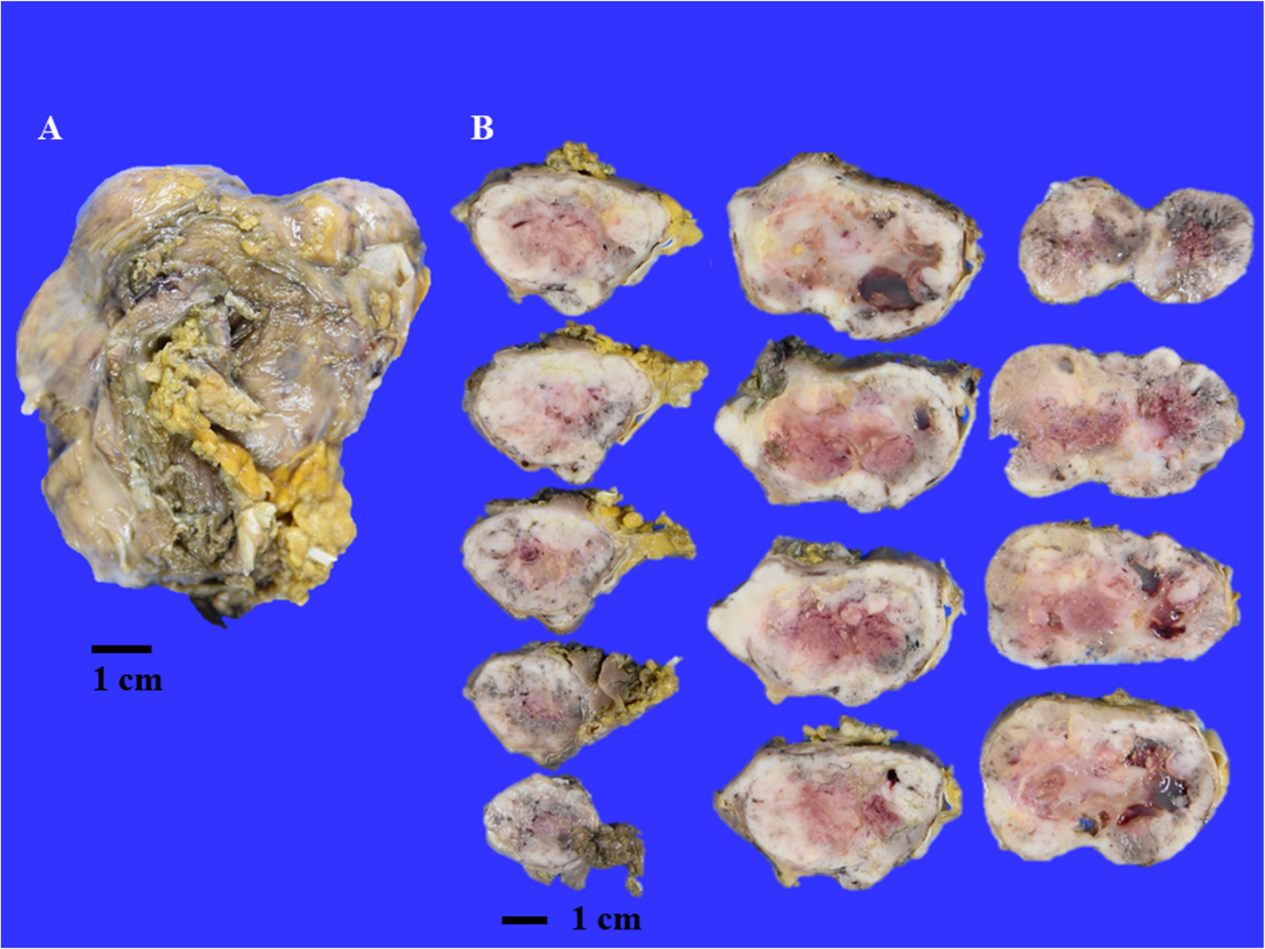
Gross image of the thyroid. **A** The thyroid weighed 110 g and was 7.6 × 6.4 × 3.0 cm in size. The thyroid was slightly hard. **B** The cut surface of thyroid showed a white solid mass with central haemorrhagic necrosis

**Fig. 5:**
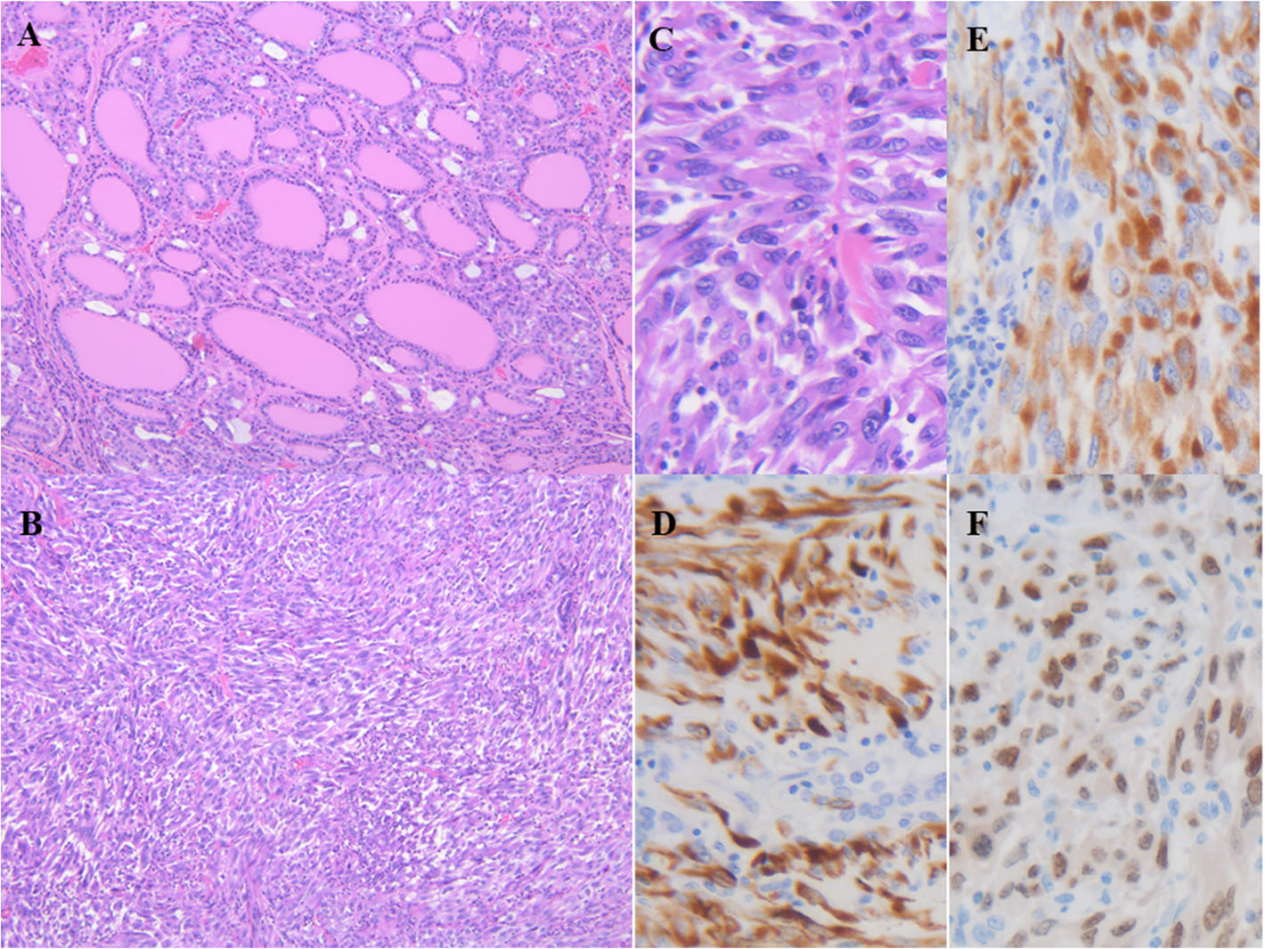
Microscopic image of the thyroid. **A** Nodules with thyroid follicles of various sizes invading the surrounding tissues were mainly observed (hematoxylin and eosin, low magnification). **B** Atypical spindle tumour cells proliferating solidly without follicles were also observed (hematoxylin and eosin, low magnification). **C** Enlargement of the image shown in B (hematoxylin and eosin, high magnification). After immunostaining, the spindle tumour cells were positive for cytokeratin AE1/AE3 (**D**), CAM5.2 (**E**) and PAX8 (**F**) (high magnification)

The thyroid was slightly hard and was weakly adhered to the trachea (Fig. 4). The tumour invaded the sternothyroid muscle, lymph nodes, and veins over the thyroid capsule. The cut surface showed a white solid mass with central haemorrhagic necrosis. The histological images revealed mainly formed nodules with thyroid follicles of various sizes invading the surrounding tissues, but they were mixed with atypical spindle tumour cells proliferating solidly without follicles (Fig. 5). After immunostaining, the spindle tumour cells were positive for cytokeratin AE1/AE3, CAM5.2 and paired box gene 8 (PAX8) and negative for epithelial membrane antigen (EMA), Tg, carcinoembryonic antigen (CEA), thyroid transcription factor-1 (TTF-1), and p53. Based on these findings, she was diagnosed with anaplastic thyroid carcinoma (ATC) arising from follicular thyroid carcinoma (FTC).

### <Other organs>

**Fig. 6:**
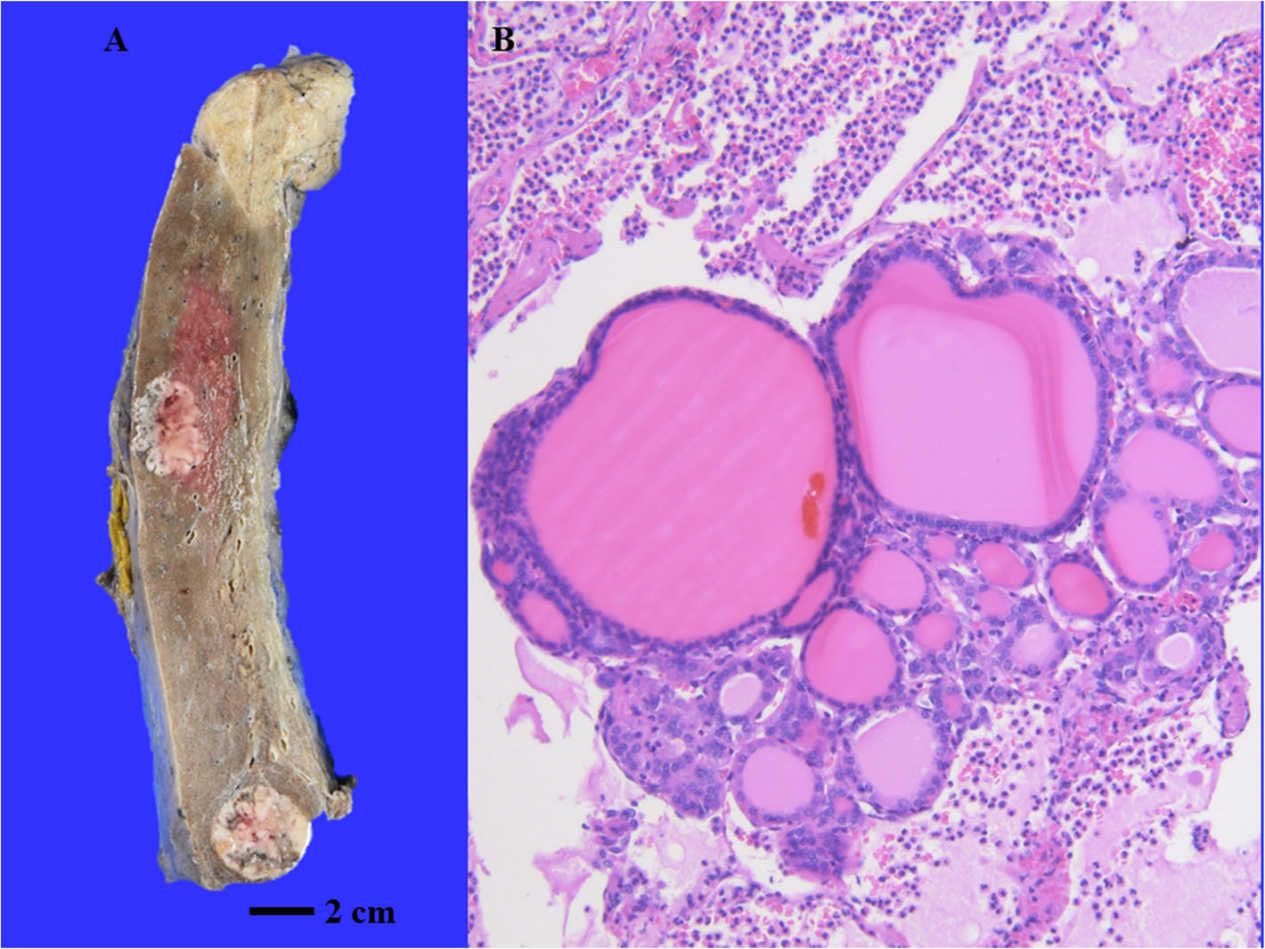
Pathological findings of left lung. **A** There were well-defined white lesions in left lung. **B** Follicles of various sizes were observed and were considered to be metastases of FTC. No anaplastic cancer tissue was found in the metastatic lesions, which all showed findings of FTC (hematoxylin and eosin, low magnification)

The FTC filtrated the trachea. There were well-defined white lesions in both lungs, which were histologically metastases of FTC (Fig. 6). In addition, metastases were also observed in the hilar and superior mediastinal lymph nodes. No anaplastic cancer tissue was found in the metastatic lesions, which all showed findings of FTC. The ascending colon cancer that had been found before her death was moderately differentiated tubular adenocarcinoma invading the subserosal tissue. However, the histological and immunohistochemical features of this tumour were completely different from those of thyroid cancer. No infarction in the cardiac wall or obstruction in the coronary arteries was observed.

## Discussion and conclusions

We reported a case of thyroid crisis likely caused by metastatic thyroid cancer. Multidisciplinary conservative treatment was not effective for her illness, and she died on the fifth day of hospitalization. The autopsy revealed the presence of primary ATC arising from FTC and multiple FTC metastases.

The Japan Thyroid Association has proposed diagnostic criteria for thyroid crisis [2]. The presence of thyrotoxicosis with elevated levels of FT3 or FT4 is a prerequisite for diagnosis. Patients with thyroid crisis have central nervous system manifestations, fever, tachycardia, congestive heart failure, and gastrointestinal/hepatic manifestations. Our patient displayed all of these parameters except for gastrointestinal/hepatic manifestations, so her symptoms were consistent with the diagnosis of thyroid crisis.

In Japan, the primary cause of thyrotoxicosis among patients with thyroid crisis is Graves’ disease, followed by destructive thyroiditis [1]. The potential trigger of thyroid crisis has been proven in 70% of patients. The most common trigger of thyroid crisis was the irregular use or discontinuation of antithyroid medication, and the second was infection. Other minor triggers are also known, such as diabetic ketoacidosis, severe emotional stress, trauma, surgery, radioiodine therapy, and pregnancy/delivery [1, 2] Among the causes of thyrotoxicosis, Graves’ disease was excluded as she was negative for TSH receptor antibody. She had displayed prolonged thyrotoxicosis for 2 years before hospitalization; her clinical course was not typical for destructive thyroiditis, such as subacute thyroiditis and painless thyroiditis. In addition, the FT3/FT4 ratio is lower in destructive thyroiditis than in Graves’ disease, and several studies have shown that the optimal FT3/FT4 ratio cut-off value is 2.8–3.0 [5, 6]. In the present case, the FT3/FT4 ratio was elevated at the time of the first diagnosis of overt thyrotoxicosis. These findings suggested that the thyrotoxicosis in this case was not due to destructive thyroiditis. Thus, most likely, thyroid cancer induced her thyroid crisis; however, the trigger is not completely clear.

Hyperfunctioning thyroid cancer can absorb iodine and synthesize/release thyroid hormone [4]. Activating mutations in TSH receptor genes activate the intracellular cyclic adenosine monophosphate (cAMP) cascade, which is the most considerable cause of hyperthyroidism due to thyroid cancer [7]. Among hyperfunctioning thyroid cancers, FTC has been shown to have a markedly higher prevalence (46.5% for primary and 71.4% for metastatic disease) than other types [4]. The Surveillance, Epidemiology and End Results (SEER) cancer registry (1974–2013) indicates that FTC only accounts for 10.8% of all thyroid cancers [8]. These data indicate that the proportion of FTC among hormone-producing thyroid cancers is high. Additionally, the tumour burden of hyperfunctioning thyroid cancer is remarkably larger (4.25 ± 2.12 cm) [4]. By comparison, the SEER cancer registry programme indicates that 28.6% of thyroid carcinomas are ≤1.0 cm in size, 26.0% are > 1.0 to ≤2.0 cm, 23.0% are > 2.0 to ≤4.0 cm, and 9.6% are > 4.0 cm [8]. Furthermore, in cases of metastatic hyperfunctioning thyroid cancers, tumour metastases are widespread or large [4]. Large primary or metastatic tumours synthesize excessive thyroid hormones, resulting in the onset of hyperthyroidism. In the present case of FTC, the primary tumour was large in size, and the metastases were widespread, consistent with previous reports. The patient had subclinical thyrotoxicosis at the time of the first diagnosis. Hormone production by both/either primary and/or metastatic FTC lesions would cause/later augment her thyrotoxicosis.

The effect of KI on hyperthyroidism in thyroid cancer patients has not yet been established, although a former report indicated the beneficial effects of inorganic iodine used in combination with antithyroid drugs and glucocorticoids on thyroid cancer-associated hyperthyroidism [9, 10]. KI in combination with antithyroid drugs is generally the gold standard in thyroid crisis cases [1]. However, surprisingly, little is known about whether KI is effective in thyroid cancer patients via the Wolff-Chaikoff effect. The molecular mechanisms of the Wolff-Chaikoff effect have not been established [11, 12]. Most research analysing the direct effects of iodine was performed on hypophysectomized animals given a standard dose of TSH, essentially regulating thyroid activity [13]. Therefore, the effects of KI in patients with diminished levels of either TSH or thyroid-stimulating antibody, such as the presented patient, are absolutely unknown, and we could not even obtain sufficient information from preclinical studies. Taken together, whether KI should be used in thyroid cancer patients with hyperthyroidism needs to be established by further investigations, and we may need to consider the harmful influence of iodine in cancer patients due to thyroid hormone secretion and potential crisis.

To the best of our knowledge, there have been only five reports about thyroid crisis due to thyroid cancer (Table 2) [9, 10, 14–16]. All were due to differentiated thyroid cancer, including papillary thyroid carcinoma (PTC) (one case), a follicular variant of PTC (three cases) and FTC (two cases, including our case). Metastases were found in five cases, including our case. Most patients had triggers, and three patients had Graves’ disease, which is similar to the general characteristics of patients with thyroid crisis [1]. The mortality rate was also high.

**Table 2:** Reported cases of thyroid crisis due to thyroid cancer

No.	Age	Sex	Pathology	Metastasis	Trigger	Outcome	Graves’ disease	Remarks	Refs.
1	20	F	PTC	no	1) pregnancy? 2) delivery	alive	+ propylthiouracil	two episodes of thyroid crisis 1) at 25 weeks gestation 2) at 2 weeks post-partum	14
2	71	F	FVPTC	bone, lung	contrast-enhanced CT incisional biopsy	death	+ no treatment		9
3	68	M	FVPTC	bone	total thyroidectomy	alive	+ thiamazole		15
4	66	F	FVPTC	bone, lung	total thyroidectomy	death	–		10
5	54	M	FTC	bone	burn injury surgery	death	unknown	post total thyroidectomy and treatment with radioactive iodine before 14 months	16
6	91	F	FTC ATC	bone, lung	unknown	death	–	primary ATC arising from FTC and multiple FTC metastases	our case

*PTC* papillary thyroid carcinoma, *FVPTC* follicular variant of PTC, *FTC* follicular thyroid carcinoma, *ATC* anaplastic thyroid carcinoma

Thyroid crisis associated with the presence of mixed FTC and ATC has never been reported. ATC proliferates rapidly and has a very poor prognosis [17]. There are several reports of patients with thyrotoxicosis due to ATC [18]. Compared to other subtypes of thyroid cancer, ATC rarely causes thyrotoxicosis or thyroid crisis. ATC may cause thyrotoxicosis through two mechanisms: leakage of thyroid hormone into the bloodstream via the mechanical destruction of thyrocytes, or hyperfunctioning metastatic tumours [19, 20]. In the present case, the patient’s symptoms rapidly deteriorated in the last 2 weeks before admission. During the clinical course, Tg peaked 6 months before admission then decreased, and her urinary iodine excretion was very high on admission. These data suggest that the ATC developed from the transformation of the coexisting FTC. Fozzatti et al. showed that the secretome of cancer-associated fibroblasts activated by ATC cell-derived conditioned media promoted FTC proliferation and invasion [21]. Based on this report, ATC facilitated FTC proliferation and metastases in the present case, promoting thyrotoxicosis severity.

In summary, we reported a case of thyroid crisis due to metastatic thyroid cancer. The autopsy revealed the presence of ATC arising from FTC and multiple metastases composed of FTC. Her thyrotoxicosis was caused by both/either primary and/or metastatic FTC lesions and perhaps facilitated by ATC. Although the trigger of her thyroid crisis was not identified, the aggravated progression of her clinical course suggests that careful monitoring of thyroid hormones and appropriate intervention are essential for the management of patients with thyroid cancer.

## Data Availability

The datasets used and/or analysed during the current study are available from the corresponding author on reasonable request.

## Abbreviations

CT: Computed tomography
TSH: Thyroid-stimulating hormone
FT4: Free thyroxine
Tg: Thyroglobulin
FT3: Free triiodothyronine
KI: Potassium iodide
HCG: Human chorionic gonadotropin
PAX8: Paired box gene 8
EMA: Epithelial membrane antigen
CEA: Carcinoembryonic antigen
TTF-1: Thyroid transcription factor-1
ATC: Anaplastic thyroid carcinoma
FTC: Follicular thyroid carcinoma
cAMP: Cyclic adenosine monophosphate
PTC: Papillary thyroid carcinoma
